# Insights into the Role of Oxidative Stress and Reactive Oxygen Species in Parasitic Diseases

**DOI:** 10.3390/antiox12051010

**Published:** 2023-04-27

**Authors:** Serge Ankri

**Affiliations:** Department of Molecular Microbiology, Ruth and Bruce Rappaport Faculty of Medicine, Technion, Haifa 31096, Israel; sankri@technion.ac.il

Parasitic infections remain a significant public health challenge in many parts of the world, especially in developing countries. Despite significant progress in their treatment, these diseases often cause long-term illness, disability, and mortality. Parasites face various challenges within the host, including oxidative and nitrosative stress generated by the host’s immune response. Understanding how parasites respond to these challenges is crucial to our comprehension of parasite–host interactions at both the cellular and molecular levels. This Special Issue includes articles on various protozoan, helminth, and arthropod parasites that highlight recent advances in our understanding of the role of oxidative stress, nitrosative stress, and metabolic pathways in parasitic diseases. These papers cover critical topics, such as the response and adaptation of parasites to oxidative stress induced by drugs [1,2]; their localization in the host during their life cycle [3]; or by nutrition [4], plant-based, and probiotic approaches to modulating parasites’ redox responses [5,6], as well as redox talk between parasites and the host’s immune defense cells [7,8,9]. Additionally, this Special Issue addresses nitric oxide resistance by discussing the role of glucose consumption and GSH-mediated redox capability in the resistance of Leishmania to nitrosative stress [10]. These examples underscore the importance of understanding the mechanisms underlying parasitic diseases, and lay the foundation for the development of innovative treatments targeting parasites’ defense mechanisms against oxidative stress. I hope that this special edition will serve as a valuable resource for researchers, students, and physicians studying parasitic infections. Finally, I would like to express my gratitude to all the authors who contributed to this Special Issue, and to the *Antioxidants* team for their assistance during the review and editorial process.
